# Setting development goals using stochastic dynamical system models

**DOI:** 10.1371/journal.pone.0171560

**Published:** 2017-02-27

**Authors:** Shyam Ranganathan, Stamatios C. Nicolis, Ranjula Bali Swain, David J. T. Sumpter

**Affiliations:** 1 Department of Statistics, Virginia Polytechnic Institute and State University, Blacksburg, VA, United States of America; 2 Service de Chimie Physique et Biologie Théorique, Faculté des Sciences, Campus Plaine, C.P. 231, Université Libre de Bruxelles, Brussels, Belgium; 3 Mistra Centre for Sustainable Markets, Stockholm School of Economics, and Södertörn University, Stockholm, Sweden; 4 Department of Mathematics, Uppsala University, Uppsala, Sweden; University College London, UNITED KINGDOM

## Abstract

The Millennium Development Goals (MDG) programme was an ambitious attempt to encourage a globalised solution to important but often-overlooked development problems. The programme led to wide-ranging development but it has also been criticised for unrealistic and arbitrary targets. In this paper, we show how country-specific development targets can be set using stochastic, dynamical system models built from historical data. In particular, we show that the MDG target of two-thirds reduction of child mortality from 1990 levels was infeasible for most countries, especially in sub-Saharan Africa. At the same time, the MDG targets were not ambitious enough for fast-developing countries such as Brazil and China. We suggest that model-based setting of country-specific targets is essential for the success of global development programmes such as the Sustainable Development Goals (SDG). This approach should provide clear, quantifiable targets for policymakers.

## 1 Introduction

Initiated in 2000, the MDG programme addressed eight major areas of concern for developing and under-developed countries identified by the United Nations. Based on an understanding of human development from the seminal works of [1] and [2] among others, the MDG programme was built on similar historical ventures [3]. Countries came together to commit resources towards reducing extreme poverty by half, child mortality by two-thirds etc before 2015 from their baseline values in 1990.

The philosophy behind the MDGs and the goal-setting has been criticised for various reasons [4–6]. For instance, rights-based campaigners have pointed to the inadequacy of the MDG framework to address key issues in development as it ignores imbalances and inequalities within countries [7]. A more general question has also been raised as to whether having quantitative goals without addressing causative questions is useful or if they might even be counter-productive to overall human development in the long run [8]. Quite apart from the theoretical questions raised, it is important to evaluate if the MDG targets were reasonable even within its own framework.

A specific criticism in the case of socio-economic goals like reducing child mortality is based on the fact that, in spite of billions of dollars of aid pouring in to help developing countries [9], a number of them have not been able to achieve the targets set [10]. As a bloc, sub-Saharan countries have regressed relative to the rest of the world from the baseline year of 1990. In 1990, 30.4% of all child deaths occured in sub-Saharan Africa, whereas in 2015, an estimated 49.6% of all child deaths happened in the region [11].

This widening of the gap is partly because of the tremendous improvements made in the rest of the world, but researchers have argued that this is also partly because the goals set up countries in sub-Saharan Africa for failure [4, 12]. The global targets did not distinguish between regional and socio-economic differences in development trajectories and hence the unrealistic targets could not be met just by expending more resources.

One suggestion to avoid judging African (or under-developed countries in general) too harshly has been to look at progress towards reaching the target rather than actual indicator variable levels [13]. But this makes the MDG targets aspirational without direct value for national planning. We suggest instead that the targets themselves must be set based on the available data in which case progress towards reaching the target is an actual measure of improvement in the country’s performance. This has also been addressed to an extent in literature (see [13] for instance).

The dynamics of development is considered in multiple papers to evaluate how countries perform with respect to the MDGs [14, 15]. In this paper, we generalize this approach by selecting the best from a large set of potential models, which implicitly include the basic linear, and other non-linear models studied in previous literature among them. Also, we explicitly model the dynamics in development by using yearly changes in the indicator variable for the development target based on historical data.

We illustrate our methodology using the MDG target of reducing child mortality in all countries by two-thirds from their 1990 levels before 2015. In 2000, when the goals were set, a major cause for concern was that there were still many countries in sub-Saharan Africa where over ten per cent of all children were likely to die before age five. While there has been significant improvement with a 53.5% decrease in global child mortality levels by 2015 [11], most countries in this region (and 119 of the total 169 in our dataset) did not reach their target.

Various theoretical and empirical studies show how child mortality is affected by the fertility rate and GDP in a country [16–20]. In a previous paper we built a complex model of the demographic transition that accounted for the interactions between these three key socio-economic indicator variables [21].

We showed that child mortality decline is increased by the level of the country’s GDP, which increases faster when the fertility rate is lower. The fertility rate, in turn, decreases faster when the child mortality is low, thus setting up a virtuous cycle that is observed as the demographic transition. The changes in child mortality are not directly affected by the fertility rate. Since we are focusing on the MDGs, in this paper, we build a dynamical system model that captures changes in child mortality as a function of itself and GDP as specified in the fertility decline model, and specify changes in GDP as a function of child mortality and GDP alone, ignoring the fertility rate variable for present purposes. Note that adding more predictor variables will only strengthen the case for model-based country-specific targets that we advocate in this paper. Also, as mentioned in [21], adding more predictor variables to our modeling methodology is easily achieved.

Based on our model (built using data up to 2000), we predict child mortality trajectories for countries from 2000 to 2015. These are validated against the data available for countries between 2000 and 2015. Using these trajectories, we quantitatively demonstrate that the MDG targets were set without due regard to historical trajectories for vulnerable countries, thus setting them up for failure as argued by others [4, 12].

We present a simple alternative for setting the child mortality targets. Using our model trajectories, a baseline target-setting approach would be to take the predicted 2015 levels from our model as the targets. This baseline target captures the average performance of countries in a “business-as-usual” scenario. Using the standard errors generated from model simulations, a more ambitious target can also be set. This allows us to balance the reasonableness of setting a feasible target with an attempt to achieve better than business-as-usual performance.

Development policymakers have started moving on from MDGs to the Sustainable Development Goals (SDGs), but setting more realistic targets is still an important consideration for the agencies, and our method provides them a good tool for that purpose. While demonstrating that qualitative criticism of the MDG targets is justified, we provide an easily generalisable methodology that can provide country-specific, quantitative targets based on realistic prediction scenarios.

## 2 Materials and methods

### 2.1 Model

We define a dynamic model of child mortality in terms of yearly changes in child mortality as a function of the levels of child mortality and other covariates in the previous years.

ΔC(i,t)=f(C(i,t),x(i,t),y(i,t)...),i=1,...,N;t=1,...,T (1)

where *C*(*i*, *t*) is the child mortality level for country *i* at time *t*, Δ*C*(*i*, *t*) = *C*(*i*, *t* + 1) − *C*(*i*, *t*) is the yearly change from the level at time *t* and *x*, *y*, … are covariates that predict changes in child mortality. Thus, we assume in this model that changes in child mortality are due to the levels of the state variables *C*, *x*, *y*, … etc. The model function *f*(.) defines the structure of this influence by specifying how the change in child mortality is related to these state variables. Using this dynamic model, and the values of *C*(*i*, *t*), *x*(*i*, *t*), … for *i* = 1, …, *N*;*t* = 1, …, *T* in the model function *f*(.) we can predict future values of *C*(*i*, *t*), *t* = *T* + 1, … if we also get good predictions of *x*(*i*, *t*), *y*(*i*, *t*), for *t* = *T* + 1, ….

In an earlier paper [22], we used polynomial basis functions *f*(*x*, *y*, …) = *a*_0_ + *a*_1_ *x* + *a*_2_ *y* + … + *a*_*k*_ *xy* + … which included linear effects as well as non-linearities and interaction terms between the variables to construct dynamical system models for socio-economic systems. In this method, we use ordinary differential equations (the yearly changes can then be thought of as yearly samples from the continuous time variables) to model the changes in indicator variables. In a subsequent paper [21] we showed how changes in child mortality can be accurately modeled using log GDP per capita and fertility rates as indicator variables. In this paper, we use the same methodology to construct a simple two-variable dynamical system model using data on child mortality (*C*) and log GDP per capita (*G*). The actual modeling methodology is described below.

The full specification of the two-variable difference equation model for predicting changes in child mortality is of the form

$$\begin{array}{ccc} \Delta C & = & a_{0}+a_{1}C+a_{2}G+a_{3}C^{2}+a_{4}G^{2}+\frac{a_{5}}{C}+\frac{a_{6}}{G}+a_{7}CG+a_{8}\frac{C}{G}+a_{9}\frac{G}{C}+\frac{a_{10}}{CG}+\frac{a_{11}}{C^{2}}+\frac{a_{12}}{G^{2}}\\ \Delta G & = & b_{0}+b_{1}C+b_{2}G+b_{3}C^{2}+b_{4}G^{2}+\frac{b_{5}}{C}+\frac{b_{6}}{G}+b_{7}CG+b_{8}\frac{C}{G}+b_{9}\frac{G}{C}+\frac{b_{10}}{CG}+\frac{b_{11}}{C^{2}}+\frac{b_{12}}{G^{2}} \end{array}$$

where we have used *C* and *G* to denote *C*(*i*, *t*) and *G*(*i*, *t*) as defined above.

We have yearly data on *C* and *G* and we estimate the model coefficients using multiple regression based on the equations above. Since we need the most parsimonious description of the relationships, we select the best sub-model from this full model specification using the two-stage algorithm described in the previous paper. First we find the log-likelihoods of all sub-models and choose only the models that have the highest log-likelihood value given a specified number of terms. We call these *M*_1_, *M*_2_, …, *M*_*i*_, … where *i* is the number of polynomial terms in the model. In the second step, we choose the best model from among *M*_1_, *M*_2_… by calculating the Bayes factor [22] for these models and choosing the model with the highest Bayes factor value. The Bayes factor applies a penalty to models with more terms and hence ensures parsimony in model specification. This is necessary because the likelihood function is monotonic in number of terms and a model with more terms will have higher likelihood value but may fit artifactual patterns due to noise. Using the Bayes factor applies a penalty to models with more terms and typically the best model is the one with a sufficiently high log-likelihood value that avoids the diminishing returns effect of adding too many terms to capture insignificant amounts of information. It is useful to note that the Bayes factor is a more general version of the Bayesian information criterion (BIC) under certain assumptions as noted in [22], [23].

Using this methodology, we get a two-variable system that provides information on how the system would behave in the presence of noise. We characterise the noise in this dynamical system based on the residual errors in the fitting and obtain equations for changes in the variables for each country *i* at each time *t* of the form

$$\begin{array}{ccc} \Delta C(i,t) & = & f\left(C\left(i,t\right),G\left(i,t\right)\right)+\epsilon_{C}\left(i,t\right) \end{array}$$ (2)

$$\begin{array}{ccc} \Delta G(i,t) & = & g\left(C\left(i,t\right),G\left(i,t\right)\right)+\epsilon_{G}\left(i,t\right) \end{array}$$ (3)

Using this stochastic equation, we can predict the future values of *C*(*i*, *t*) and *G*(*i*, *t*) for different countries *i* based on their initial conditions as specified from the data. To validate this approach, we start with the values of *C* and *G* in 2000 for all countries and integrate them forward with many realisations.

As seen from data, child mortality decreases are heteroskedastic and tend to be more noisy when child mortality is high and less so when it is low. The error variable is given by *ϵ*_*C*_(*i*, *t*) = *αn*_1_(*i*, *t*) + *βC*(*i*, *t*)*n*_2_(*i*, *t*), where *n*_1_(*i*, *t*), *n*_2_(*i*, *t*) are standard normal variables identically distributed as *N*(0, 1) and independent across both country index *i* and time index *t*. *α*, *β* are the error standard deviation parameters and *C* is the value of child mortality. Changes in *G* are homoskedastic (as seen from the data and tested later). The error variable for changes in *G* is *ϵ*_*G*_(*i*, *t*) = *γn*_3_(*i*, *t*), where *n*_3_(*i*, *t*) are standard normal variables distributed as *N*(0, 1) and independent across both country index *i* and time index *t*, and *γ* is the noise standard deviation parameter.

Using this error variable model allows us to create a set of possible trajectories for all countries from 2000 − 2015 and calculate the mean of these trajectories and standard errors to quantify the effect of noise on the models. Note that heteroskedasticity in the child mortality model implies that the OLS estimates, while unbiased, are not efficient. Hence, robust (heteroskedaticity-consistent) standard errors need to be used instead of the standard errors as calculated from the OLS estimator (for instance, see [24]). In this paper, we do not use the OLS standard errors but instead use Monte Carlo simulations of the error variable (with heteroskedasticity specified in parametric form) to compute the standard errors. This ensures that our standard errors for changes in C and G are robust and heteroskedasticity-consistent.

We use these models to set development targets. A “baseline” target is defined as one where the country is only expected to achieve child mortality values as predicted by the deterministic dynamical system. This corresponds to a business-as-usual scenario. An “ambitious” target is defined as the lower end of the error bars. For instance, if C*(*i*, *T*) is the predicted child mortality level for country *i* time *T* and σ*(*i*, *T*) is the estimated error standard deviation at the same time for country *i* based on our model, the baseline target will be C*(*i*, *T*) and the ambitious target will be C*(*i*, *T*) − 2σ*(*i*, *T*).

Even more ambitious targets can be set based on specific inputs and policy decisions on spending for the goals. Since our models explicitly model the effect of *G* on *C*, any anticipated additional spending to achieve the *C* target can be thought of as a shock on the *G* variable affecting only the *C* equation without systematically altering the economy (and hence the G-system) as a whole. We discuss this later in the paper.

### 2.2 Data

We use the child mortality and GDP per capita (in PPP dollars) indicator variables from the World Development Indicators dataset published by the World Bank. We use data from 1960–2015 for 169 countries from the dataset.

Child mortality is measured in number of children lost before age 5 per 1,000 live births and, in the dataset, ranges from 2 to 327. In this paper we denote it by *C*. The GDP per capita (in PPP dollars) is a standard measure for the economic output of a country. We use the logarithm of the data values and in the log scale, the data ranges from around 5 to 12. We denote this variable by *G*. From the *C* and *G* values, we find the yearly changes in the indicator variables for each country by taking successive differences and denote these change variables by Δ*C* and Δ*G*.

**Fig 1:**
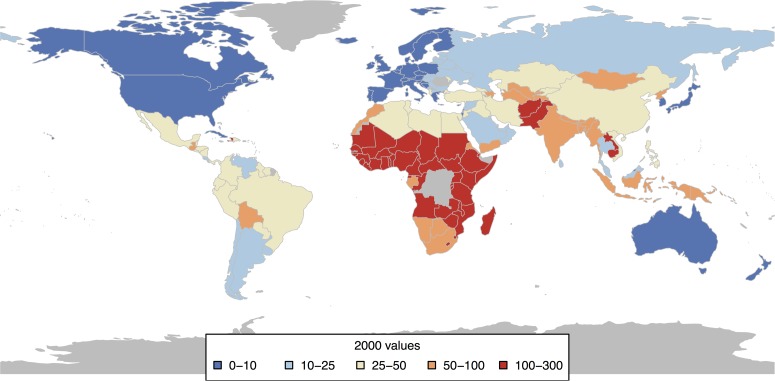
2000 child mortality values for countries (under-5 mortality per 1,000 live births).

Fig 1 shows the state of the world in terms of child mortality in 2000, when the MDGs were adopted. Many countries in sub-Saharan Africa had child mortality levels between 100 and 300, which is much higher than the threshold for a “high-mortality” country which is defined as a country with child mortality level above 40 [11].

Though we also show developed countries in this map and later in the paper, we note that the MDGs were specifically targeted towards developing countries and their success or failure needs to be evaluated based only on the performance of these countries. At the same time, it is interesting to see the performance of the developed countries relative to the MDG targets since this helps validate our approach of model-based setting of development targets, especially in the upcoming SDG programme.

**Fig 2:**
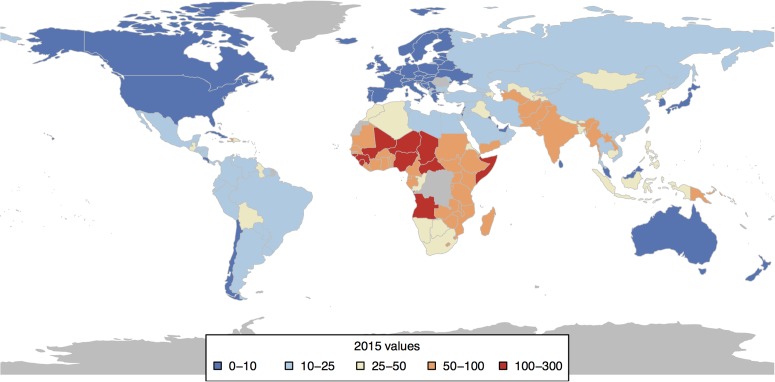
2015 child mortality values for countries (under-5 mortality per 1,000 live births).

As seen from Fig 2, just about every country made a significant improvement by 2015. The global child mortality measured in terms of number of children lost actually fell by 53.5% from its 1990 levels of around 12.7 million to 5.9 million in 2015 [11].

## 3 Results

### 3.1 Estimated model

**Fig 3:**
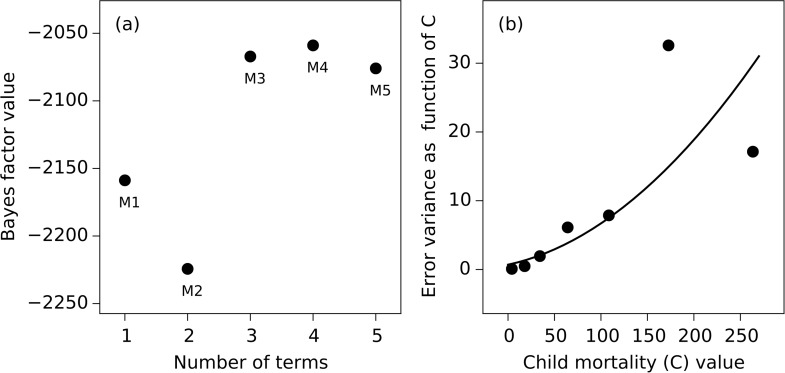
(a) Bayes factor values for dynamical system models with different number of terms. The four term model M4 is the best fit model. In this paper we use the 3 term model M3 which performs close to M4 for computational ease. (b) Model fitting error variance as a function of *C*. The blue dots shows the residual error standard deviation based on the model fitting and the black curve shows a linear regression model of the standard deviation fit to the data.

We estimate the best fit model with data for all countries from 1960 up to 2000, the year when the MDGs were announced. Thus our model only uses the data until the time when the policy-makers set the MDG targets. The model that best captures the relationship between child mortality (*C*) and log GDP per capita (*G*) based on data up to 2000 is given by (Fig 3a)

$$\begin{array}{ccc} \Delta G & = & \frac{-16.8}{C}+\frac{95.7}{GC}+\frac{0.732G}{C}+\epsilon_{G} \end{array}$$ (4)

$$\begin{array}{ccc} \Delta C & = & 0.439C-0.0354CG-\frac{1.47C}{G}+\epsilon_{C} \end{array}$$ (5)

where we have dropped the country and time indices *i* and *t* from the variables for readability. The model shows that there is an interaction between the two variables and changes in child mortality are higher when *G* is high, which itself increases faster when *C* is low. Under the constraint that we describe *G* and *C* only in terms of two variables, we see that there is a feedback effect due to the interaction terms and the presence of both variables in the change equations for each. We use this model to show that country-specific development targets can be set in a meaningful manner. Further refinements in model specification, using more explanatory variables for instance, will improve the quality of the development targets and result in better policymaking.

For instance, the adjusted *R*^2^ value for the Δ*C* model specified here is only 0.2. We show in [21] that it increases to 0.3 with the addition of the total fertility rate variable, and the addition of more explanatory variables that explain child mortality in a country will further increase the adjusted *R*^2^ value. Similarly, we do not use any of the multiple explanatory variables for GDP as studied in the growth econometrics literature resulting in a very low adjusted *R*^2^ value of 0.01 for the Δ*G* model specified above. This can be improved by specifying more complex models in future research. We emphasize, however, that even our simple models with just two explanatory variables result in significant gains in setting development targets as explained in the next subsection.

We test the data and find evidence for the presence of heteroskedasticity in child mortality. Based on the modeling errors for child mortality (Fig 3b), the noise variable *ϵ*_*C*_ has error standard deviation given by *σ*_*C*_ = 0.83 + 0.018*C*. The error variable for country *i* at time *t* is hence given by *ϵ*_*C*_ = 0.83*n*_1_ + 0.018*Cn*_2_, where *n*_1_, *n*_2_ are defined as above.

The noise variable for the *G* model can be best fitted as additive, independent Gaussian noise with variance 0.007. The noise variable is then given by *ϵ*_*G*_ = 0.086*n*_3_ where *n*_3_ is the standard white Gaussian noise variable.

### 3.2 Setting reasonable development goals

**Fig 4:**
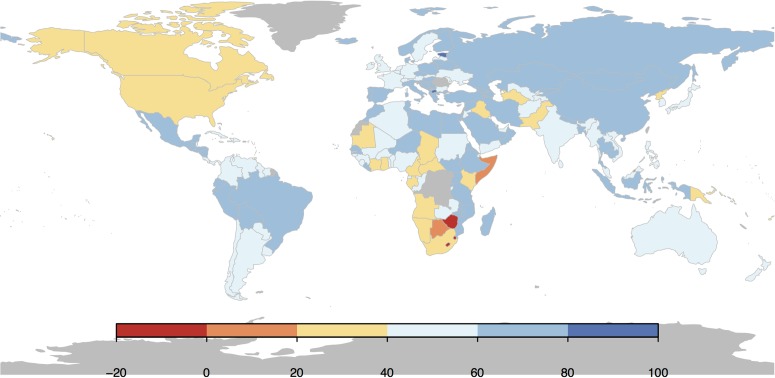
Percentage reduction from 1990 values of child mortality reduction for different countries in the world in 2015. The MDG target is a 66.7% reduction by 2015 and the map shows that there are many countries which managed only between 20 − 60% reduction. Three countries—Lesotho, Swaziland and Zimbabwe have higher child mortality values in 2015 than they had in 1990.

Fig 4 shows the percentage reduction in child mortality for countries from 1990 levels. By the timeframe of 2015 set for the MDGs, only 50 of the 169 countries we have data for have met the target. Also, nearly a fourth of them are OECD member states which already had very low child mortality levels to begin with and were not of much interest to the MDG policymakers. This raises the question as to whether there were systematic failures in helping countries achieving the MDG targets. While many of the failures have been pointed out, another important question concerns whether the targets themselves were reasonable in the first place, and if they were not, how do we set better development targets. We show how our methodology described above can be used to set good development targets and also evaluate the reasonableness of the MDG targets.

A big reason the MDG targets were criticized was because a two-thirds reduction in child mortality levels, while desirable, was not necessarily feasible for high mortality countries. Instead, we use our model based on data up to 2000 to predict the “future” levels (2001 to 2015) of child mortality of the different countries and use these to set country-specific development targets. We run 10,000 different realisations of the child mortality trajectories for each country to model different possible scenarios and compute the predicted child mortality statistics for the countries.

As we an illustration, for a country like Angola, which had a child mortality level of around 223 in 1990, this translates to a baseline target of 144 and an ambitious target of 116 both much higher than the over-ambitious MDG target of 74.55. Our targets were clearly more “reasonable” in hindsight given that various socio-economic troubles allowed Angola to reduce its child mortality level only to 157 by 2015.

**Fig 5:**
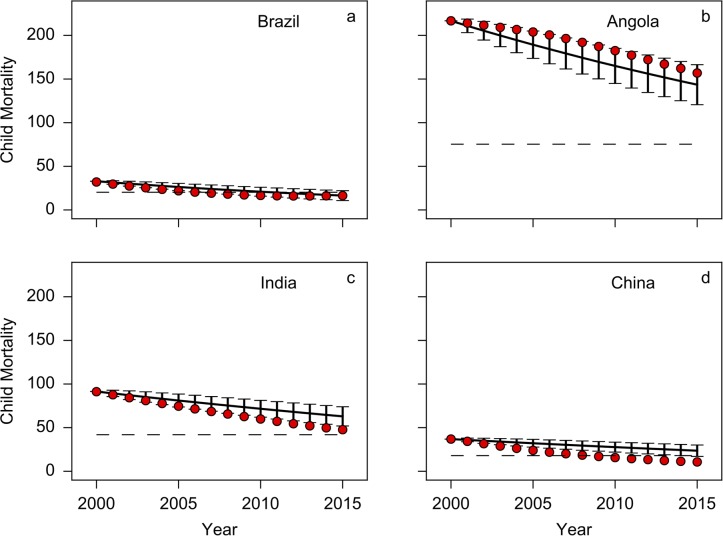
Performance of four countries based on model predictions. The red dots show the actual values of child mortality for the different countries, the dashed line shows the MDG target. The solid black line is the mean predicted trajectory of the country based on our model and 10,000 different realisations of the noise process with corresponding errors corresponding to twice the standard deviation as obtained from the simulations. Angola and Brazil are performing within confidence intervals of our model predictions. Thus these two countries are performing as would be expected by our model but while Brazil has easily met its MDG target, Angola is far from reaching its MDG target. India and China are over-performing relative to our model. India has actually not reached its MDG target whereas Brazil and China have already exceeded their MDG targets (see Model section for details).

Fig 5 shows the trajectories of four countries during the 15 years from 2000 to 2015 along with the model-predicted trajectory and standard errors (we use twice standard errors to indicate 95% confidence intervals). The figure shows that that the model predicts the performance of the different countries reasonably well in the presence of noise. Angola’s performance relative to the model (worse than the mean predictions) can be attributed to civil war (that ended in 2002) and other political issues. On the other hand, the first decade and a half of the twenty-first century saw the emergence of Brazil, India and China as major players in the global economy and sustained investments in human capital meant that they performed better than the model mean predictions.

The dashed lines in the figure also show the respective MDG target for each of the countries. It is clear that the MDG targets do not align either with the model predictions or with actual data. For Brazil and China, the MDG targets are too conservative and these two countries have indeed achieved their MDG targets with years to spare. Brazil and Angola are performing within model predictions but while Brazil has easily met its MDG target, Angola is far from reaching its MDG target. India and China are slightly over-performing relative to our model. India has actually not reached its MDG target whereas China has already exceeded their MDG targets.

On the other hand, the targets for India and Angola are too ambitious (even though India has outpaced our model-based predictions due to substantial investments in child health). As of 2015, Angola has the highest child mortality rate of 157 among all countries, nearly 4 times the threshold of 40 used to define a “high-mortality” country [11]. Given that most countries historically took a significantly longer time to reduce their child mortality from such high levels (as repeatedly noted by [12, 14] etc), it was extremely unlikely that Angola was going to even come close to its MDG target. The specific problems that the country faced in the last decade of the previous millennium and in the early 2000s only exacerbated this improbability of reaching the MDG target even further.

Table 1 extends this analysis to data from the whole world. We subdivide the countries according to a classification scheme defined by the World Bank and other world agencies. This helps us clearly see the differences in performance between countries in sub-Saharan Africa and the OECD member countries. The table shows the child mortality level in 2015, the MDG target and the baseline and ambitious targets based on our model that we have defined above. In order to help identify countries that have performed well, column values which are lower than the 2015 child mortality value for a particular country are highlighted in bold. For instance, Bahrain (ISO code BHR) has a child mortality value of 6.2 in 2015, its MDG target was 7.59 and its baseline and ambitious model targets were 4.27 and 0. Since Bahrain’s child mortality level is lower than the MDG target, the MDG column is shown in bold whereas the other two columns are shown in normal font. Thus countries which have their MDG target column in bold are those that have achieved their MDG target in 2015 and those that have their ambitious target in bold are the ones that have performed better than 95% confidence intervals for model predictions. Many of the countries that have over-performed clearly belong to sub-Saharan Africa which had the greatest thrust on investments in child mortality decline.

**Table 1:** The first column is the three letter ISO code for the country, the second column is the 2015 values of child mortality, the third column is the MDG target and the fourth and fifth columns are the baseline and ambitious targets for 2015 based on our model. A column value in bold indicates that the 2015 CM value is lower than the particular column value.

Country	CM2015	MDG	Baseline	Ambitious
**Sub-Saharan Africa**				
AGO	156.9	74.55	143.68	116.04
BEN	99.5	59.20	**107.78**	86.67
BWA	43.6	16.34	**43.77**	31.82
BFA	88.6	66.73	**141.06**	**115.60**
BDI	81.7	56.36	**114.02**	**92.41**
CPV	24.5	20.79	22.57	13.85
CMR	87.9	45.01	**106.51**	85.28
CAF	130.1	58.38	**133.64**	109.36
COM	73.5	41.38	**74.17**	57.71
COG	45.0	30.43	**76.17**	**59.16**
CIV	92.6	50.03	**98.23**	78.19
DJI	65.3	39.14	**71.29**	55.27
ERI	46.5	**49.70**	**65.43**	**50.61**
ETH	59.2	**67.65**	**111.41**	**90.58**
GAB	50.8	30.59	37.06	26.30
GHA	61.6	42.31	**71.11**	55.59
GIN	93.7	78.41	**128.81**	**104.86**
GMB	68.9	56.03	**87.83**	69.50
GNB	92.5	74.18	**136.03**	111.33
GNQ	94.1	60.72	73.59	56.56
KEN	49.4	32.57	**80.02**	62.96
LSO	90.2	28.48	83.97	66.27
LBR	69.9	**81.84**	**130.47**	**106.45**
MDG	49.6	**53.06**	**81.88**	**64.38**
MWI	64.0	**80.95**	**133.27**	**109.28**
MLI	114.7	83.89	**165.73**	**136.74**
MRT	84.7	38.87	79.38	62.22
MUS	13.5	7.62	9.15	2.94
MOZ	78.5	78.21	**128.43**	**104.73**
NAM	45.4	24.29	43.93	32.08
NER	95.5	**108.01**	**174.08**	**144.17**
NGA	108.8	70.36	**128.91**	104.31
RWA	41.7	**50.09**	**139.42**	**114.25**
SEN	47.2	46.56	**98.61**	**78.58**
SLE	120.4	88.34	**176.68**	146.66
SDN	70.1	42.24	**75.41**	58.80
SWZ	60.7	24.39	**72.65**	56.31
SYC	13.6	5.45	6.08	0.71
TCD	138.7	70.85	**145.09**	119.46
TZA	48.7	**55.11**	**99.82**	**80.42**
TGO	78.4	48.31	**90.76**	72.07
UGA	54.6	**58.97**	**112.41**	**91.10**
ZAF	40.5	20.13	39.02	28.08
ZMB	64.0	63.53	**120.25**	**97.47**
ZWE	70.7	24.62	70.36	54.66
**South Asia, Central Asia, Europe**				
BGD	37.6	**47.42**	**65.48**	**50.61**
BTN	32.9	**44.12**	**51.77**	**38.76**
IND	47.7	41.55	**62.88**	**47.96**
LKA	9.8	7.03	9.44	2.93
NPL	35.8	**46.96**	**60.13**	**45.72**
ALB	14	13.37	**15.27**	7.83
ARM	14.1	**16.40**	**19.81**	11.59
AZE	31.7	31.19	**46.91**	34.66
BGR	10.4	7.29	11.39	4.71
BIH	5.4	**6.04**	5.10	0
BLR	4.6	**5.48**	**7.72**	1.63
CYP	2.7	**3.66**	3.13	0
GEO	11.9	**15.61**	**23.02**	14.33
HRV	4.3	4.22	4.05	0
KAZ	14.1	**17.36**	**22.97**	14.45
KGZ	21.3	**21.68**	**34.48**	23.91
LVA	7.9	6.73	**8.58**	2.45
LTU	5.2	**5.45**	**5.74**	0.19
MDA	15.8	10.66	**20.74**	12.23
MKD	5.5	**12.08**	**8.56**	2.32
MLT	6.4	3.76	3.49	0
RUS	9.6	8.58	**12.45**	5.67
SRB	6.7	**9.17**	**6.77**	0.85
TJK	44.8	35.71	**70.46**	**54.89**
TKM	51.4	29.93	49.42	36.62
UKR	9	6.47	**10.79**	4.01
UZB	39.1	23.56	**43.88**	32.03
**Middle East and North Africa**				
ARE	6.8	5.45	3.05	0
BHR	6.2	**7.59**	4.27	0
DZA	25.5	15.54	20.31	12.28
EGY	24	**28.08**	**24.63**	15.81
IRN	15.5	**18.68**	**17.53**	9.97
JOR	17.9	12.11	14.95	7.63
KWT	8.6	5.51	3.60	0
LBN	8.3	**10.66**	**9.47**	3.24
LBY	13.4	**13.99**	11.56	5.22
MAR	27.6	26.63	**31.40**	21.40
OMN	11.6	**12.97**	5.85	0.76
PAK	81.1	45.74	74.59	57.91
SAU	14.5	**14.55**	8.02	2.50
TUN	14.0	**17.23**	**16.93**	9.40
YEM	41.9	41.18	**61.29**	**46.73**
**East Asia and Pacific**				
BRN	10.2	4.03	3.05	0
CHN	10.7	**17.79**	**23.71**	**14.93**
FJI	22.4	9.90	13.31	6.27
FSM	34.7	18.28	35.13	24.57
IDN	27.2	**27.82**	**30.93**	21.02
KHM	28.7	**38.77**	**82.44**	**65.11**
KIR	55.9	31.48	51.22	38.14
LAO	66.7	53.46	**82.64**	65.02
MNG	22.4	**35.61**	**41.09**	29.52
MYS	7.0	5.48	4.60	0
PHL	28.0	19.34	24.77	15.78
PLW	16.4	11.91	12.34	5.72
PNG	57.3	29.40	55.77	42.06
SLB	28.1	12.77	23.72	14.83
THA	12.3	12.24	11.66	5.00
TON	16.7	7.52	10.53	3.79
VNM	21.7	16.70	23.56	14.62
VUT	27.5	10.92	15.03	7.44
WSM	17.5	10.23	13.38	6.07
**Latin America and Caribbean**				
ARG	12.5	9.11	9.39	3.32
ATG	8.1	**8.42**	6.52	1.05
BHS	12.1	7.76	6.16	0.84
BLZ	16.5	13.07	13.97	6.82
BOL	38.4	**40.49**	**48.40**	35.74
BRA	16.4	**20.30**	**16.39**	9.04
BRB	13.0	5.97	7.48	1.70
COL	15.9	11.62	13.29	6.40
CRI	9.7	5.58	6.45	0.72
CUB	5.5	4.39	4.19	0
DOM	30.9	19.70	22.92	14.43
ECU	21.6	18.78	18.82	10.97
GRD	11.8	7.33	7.99	1.98
GTM	29.1	26.60	29.13	19.64
GUY	39.4	20.20	29.17	19.56
HTI	69.0	47.72	**75.55**	58.95
HND	20.4	19.50	**24.51**	15.46
JAM	15.7	9.83	12.51	5.71
LCA	14.30	7.46	8.99	2.78
NIC	22.10	22.04	**25.97**	16.80
PAN	17.0	10.26	13.21	6.33
PRY	20.50	15.25	19.00	11.09
PER	16.90	**26.40**	**22.53**	14.02
SLV	16.80	**19.64**	**18.43**	10.53
SUR	21.30	15.74	17.68	10.12
TTO	20.40	10.10	12.69	6.16
VCT	18.30	8.15	11.83	5.11
URY	10.10	7.62	7.86	1.91
VEN	14.90	9.74	9.76	3.56
**OECD member countries**				
AUS	3.80	3.04	2.86	0
AUT	3.50	3.14	2.66	0
BEL	4.10	3.30	2.80	0
CAN	4.90	2.74	2.82	0
CHL	8.10	6.30	5.24	0
CZE	3.40	**4.82**	3.19	0
DNK	3.50	2.94	2.74	0
EST	2.90	**6.67**	**5.29**	0
FIN	2.30	2.21	2.54	0
FRA	4.30	2.97	2.75	0
DEU	3.70	2.81	2.71	0
GRC	4.60	4.12	3.50	0
HUN	5.90	**6.27**	5.12	0
ISL	2.0	**2.11**	**2.44**	0
IRL	3.60	3.04	3.11	0
ISR	4.0	3.83	3.07	0
ITA	3.50	3.17	2.77	0
JPN	2.70	2.08	2.57	0
KOR	3.40	2.34	3.08	0
LUX	1.90	**2.90**	**2.33**	0
MEX	13.20	**15.31**	12.24	5.55
NLD	3.80	2.74	2.80	0
NZL	5.70	3.70	3.30	0
NOR	2.60	**2.87**	2.52	0
POL	5.20	**5.71**	4.44	0
PRT	3.60	**4.85**	3.31	0
SVK	7.30	5.84	5.47	0.09
SVN	2.60	**3.43**	2.88	0
ESP	4.10	3.63	3.04	0
SWE	3.00	2.28	2.48	0
CHE	3.90	2.71	2.66	0
TUR	13.50	**24.55**	**21.01**	12.90
GBR	4.20	3.07	3.00	0
USA	6.50	3.70	3.19	0

From Table 1, we see that MDG targets are too unambitious for countries that are expected to do well by the model such as Brazil and China (as substantiated by the data) and these countries have already achieved their MDG targets. The MDG targets are also too ambitious for countries that have very high child mortality. Even though many countries in sub-Saharan Africa have actually achieved the baseline prediction that is based on historical data, only 9 out of the 45 countries have achieved their MDG target and most are not close to achieving it before the end of 2015. High-mortality countries such as Burkina Faso, Malawi and Niger have performed even better than ambitious model estimates. This is both an encouraging sign for these countries and for the modeling approach to setting development targets—these countries saw heavy investment since 2000 in reductions in child mortality and efficiently reduced their child mortality levels. Since the model did not include any information on future investments (it uses only data until 2000), it could not foresee this improvement. But the model can easily be extended to include tuning parameters to capture possible policy changes that result in a faster decrease in child mortality levels.

**Fig 6:**
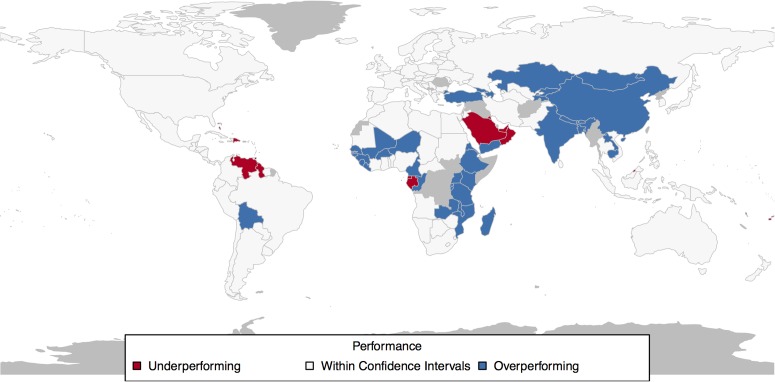
Performance of countries in 2015 based on model predictions. Red shaded countries “underperformed”—their actual child mortality level in 2015 was at least 2 standard deviations more than the model predicted average. Blue shaded countries “over-performed”—their child mortality in 2015 was at least 2 standard deviations less than the model predicted mean. White shaded countries had actual child mortality values within 2 standard deviations away from the model predicted mean. (Countries shaded grey did not have sufficient data for the simulation).

As seen from Table 1 and from Fig 6, an efficient way to set development targets would be to use the model-based approach and let policy-makers specify the level of ambition in terms of planned investments in development. In Table 1, we define “baseline” targets as the model-predicted value of child mortality for a given country. This corresponds to average or business-as-usual performance. The “ambitious” target corresponds to the lower end of the error bar (lower limit of the 95% confidence interval) and it depends on both the model-predicted mean and the standard error in the model for that country at that time instant. For a country to achieve the ambitious target, a sustained improvement much higher than historical performance for the country is required.

Our model can also account for specific and targeted increases in development spending through aid inflows etc. because the quantum of increased investment in child mortality can be directly substituted in Eq 5 to quantify the change in child mortality due to this increased investment. Specifically, we see that an increased investment of *G*_0_ dollars (per capita and measured in the log scale) can cause a minimum excess fractional decrease in child mortality given by

$$\frac{\Delta C}{C}=-0.0354G_{0}+1.47\left(\frac{1}{G}-\frac{1}{G+G0}\right)$$ (6)

This is obtained by substituting *G* + *G*_0_ in Eq 5 in place of *G*. Since fractional change in *C* is given by $\frac{\Delta C}{C}$, the additional change due to the increased investment *G*_0_ is given by Eq 6. Note that this is a lower bound on the fractional decrease because our use of *G*_0_ in Eq 5 assumes that the increase in *G* is an increase in the overall GDP per capita for the country whereas *G*_0_ is a targeted investment in child mortality decrease.

## 4 Discussion

To validate the methodology, we compare model predictions with actual country trajectories from the data. As before, we run multiple simulations of our model from year 2000 values with random noise realisations based on the noise model described above and create different possible trajectories for each country. We estimate the model predicted mean and standard deviations for each time instant from 2000 to 2015 using this method.

Fig 6 shows that the child mortality levels for most countries in 2015 lie within 95% confidence intervals for the model (shaded white in the figure). That is, most countries had actual child mortality values within 2 standard deviations from the model predicted mean values. This suggests that the model tracks country behaviour with reasonable accuracy.

Our model was built on historical data upto 2000. Hence it did not contain any information on the increased investments in child mortality and other socio-economic development indicators as a result of the MDG programme. The fact that the “over-performing” countries are also the ones which focused their efforts (possibly with the help of donor aid) on healthcare issues subsequent to the MDG announcement also helps to validate the general approach we take here to development goal-setting.

For instance, a recent UNAIDS report [25] noted that six countries—Liberia, Madagascar, Malawi, Rwanda, Togo and Zambia—had achieved the target of allocating 15% of public spending to healthcare set in the Abuja declaration of the African Union in 2001. Among these countries, only Togo did not beat the model predictions while the best-performing country, Malawi, has managed to reduce its child mortality value by 74% from 244 in 1990 to 64 in 2015, which is lower than both the MDG target and the ambitious model target that we set.

Thus far, we have shown the methodology allows us to set country-specific and hence country-calibrated development targets. The same methodology can also be used as a tool to evaluate policy decisions. For instance, we can ask if the MDG targets for child mortality were reasonably set, a question that has been raised repeatedly over the last decade by researchers focusing on sub-Saharan Africa. To answer this question, we use the same Monte Carlo simulations approach.

We run 10,000 simulations for each country starting from 2000 values of child mortality and estimate the probability of a country reaching its MDG target by 2015. This is defined as the proportion of realisations in which the country manages to get its child mortality levels below the MDG target in the presence of random noise events. A series of favourable random noise occurrences can push the country to seemingly over-perform while a series of unfavourable occurences may push it to under-perform. On average, these will cancel out and the average of all these trajectories will correspond to the model trajectories with the corresponding deviations from the mean as discussed above.

Thus the probability of reaching the MDG target measures the probability that a series of favourable random noise occurrences will allow the country to achieve the MDG target given the noise model estimated from historical data. This is an under-estimation of the actual probability since the noise model is likely to favour over-performance when there is a concerted effort to reduce child mortality levels. Hence we use a low number to measure improbability of achieving the MDG target, i.e., we assume a country was unlikely to achieve the MDG target if its estimated probability is 0.2 or lower.

**Fig 7:**
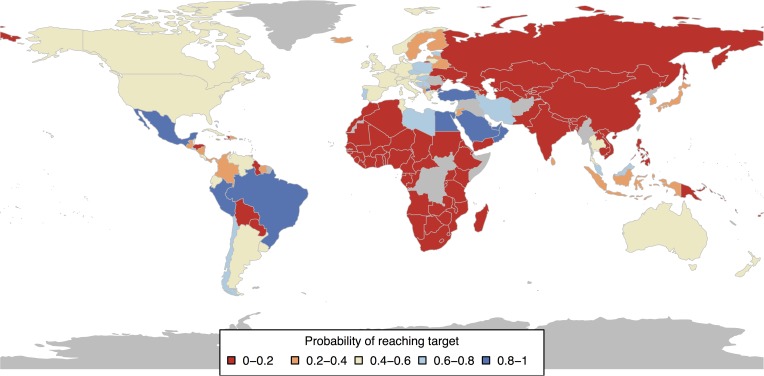
Probability of reaching the MDG target for different countries based on our model starting from 2000 values. Red indicates that a country had less than 20% chance of achieving the MDG target based on historical data. A few countries like Brazil, Mexico, Turkey and Egypt actually had a very good probability of reaching the MDG target and our models show that the MDG target was unambitious with respect to these countries.

Fig 7 shows that only a handful of countries, including Brazil, Egypt, Mexico and Turkey, had a high probability of reaching the target (many of the countries in this list actually surpassed their targets by 2013).

Most countries had an estimated probability of between 0 − 0.2, the threshold for improbability we had discussed earlier, of reaching the target based on our models. This is in line with the performance of these countries over the previous years and their distance from the MDG target in 2015. Fig 7 clearly shows that it was improbable for most countries to achieve the MDG target based on historical data and the 2000 levels. According to our evaluation, the MDGs were set in an unreasonable and arbitrary manner.

We can further validate our approach to setting the targets in this manner. For instance, only 9 out of 45 countries from sub-Saharan Africa (Table 1) reached the MDG target suggesting that the MDG targets were too ambitious. At the same time, 34 countries reached the baseline target based on our model. This indicates that the baseline target is a benchmark for average performance, as it is intended to be the target measuring “business-as-usual” performance.

The ambitious target may be used by policy-makers for countries where feasible and an even more ambitious target based on specific investments for child mortality reduction can be set based on Eq 6.

Our methodology does not use the most accurate model of how child mortality changes for the simple reason that we ignore many other important covariates. However this parsimonious model captures the essential features of the historical data in child mortality as shown in our model validation. A simple extension of this methodology to include more complex models can be made easily and we can use that model to set even better development goals that are aligned closely with the development trajectories of different countries. This will be essential to future programmes and work on developing efficient models that predict country trajectories will prove useful to the policymaker. But, in this paper, we have shown that even the use of a simple model can accurately track important trends in development trajectories and predict future development levels with reasonable accuracy, providing the policymaker with an effective tool.

## 5 Conclusions

In this paper, we have shown that the criticism of the MDGs as arbitrary by experts in development studies is justified using a statistical model of the decline in child mortality. We use this model to suggest a better approach to setting development targets based both on historical trends and possible policy goals. Setting such reasonable and country-specific development targets is key to ensure that development efforts are not wasted on infeasible goals. We believe this is especially key in the coming round of the ambitious Sustainable Development Goals in.

While we provide a parsimonious model to justify our claims, in a multi-dimensional programme like the SDGs, more complex models are required that account for a number of explanatory variables and the interactions between them. In this paper, we have provided the basic framework using which more complex models can be constructed to address those needs. This model-based policy analysis can provide a strong complement to expert knowledge in development to provide realistic development progress.
